# Why I Can’t, Won’t or Don’t Test for HIV: Insights from Australian Migrants Born in Sub-Saharan Africa, Southeast Asia and Northeast Asia

**DOI:** 10.3390/ijerph16061034

**Published:** 2019-03-21

**Authors:** Corie Gray, Roanna Lobo, Lea Narciso, Enaam Oudih, Praveena Gunaratnam, Rachel Thorpe, Gemma Crawford

**Affiliations:** 1 Collaboration for Evidence, Research and Impact in Public Health, School of Public Health, Curtin University, Kent Street, Bentley, WA 6102, Australia; roanna.lobo@curtin.edu.au (R.L.); g.crawford@curtin.edu.au (G.C.); 2 Communicable Disease Control Branch, Public Health and Clinical Systems, Department for Health and Wellbeing, Government of South Australia, Adelaide, SA 5000, Australia; lea.Narciso@sa.gov.au; 3 PEACE Multicultural Services, Relationships Australia South Australia, Adelaide, SA 5000, Australia; E.Oudih@rasa.org.au; 4 The Kirby Institute, University of New South Wales, Sydney, NSW 2052, Australia; praveenagunaratnam@gmail.com; 5 Australian Research Centre in Sex, Health and Society, La Trobe University, Bundoora, VIC 3086, Australia; R.Thorpe@latrobe.edu.au

**Keywords:** migrants, sexual health, HIV, HIV testing

## Abstract

People born in sub-Saharan Africa and Southeast Asia are overrepresented in HIV notifications in Australia. Just under half of all notifications among people from sub-Saharan Africa and Southeast Asia are diagnosed late. Increased HIV testing among these communities is necessary to ensure early diagnosis, better care and reduce likelihood of HIV onward transmission. Recently, Australia has made new HIV testing methods available: rapid HIV testing and self-testing kits. We conducted 11 focus groups with 77 participants with people from sub-Saharan Africa, Southeast Asia and Northeast Asia in four jurisdictions in Australia. Focus groups discussed barriers to HIV testing and the acceptability of new testing methods. Barriers to HIV testing included: cost and eligibility of health services, low visibility of HIV in Australia, HIV-related stigma, and missed opportunities by general practitioners (GPs) for early diagnosis of HIV and linkage into care. Participants had low levels of knowledge on where to test for HIV and the different methods available. Diverse opportunities for testing were considered important. Interventions to increase HIV testing rates among sub-Saharan African, Southeast Asia and Northeast Asian migrants in Australia need to be multi-strategic and aimed at individual, community and policy levels. New methods of HIV testing, including rapid HIV testing and self-testing, present an opportunity to engage with migrants outside of traditional health care settings.

## 1. Introduction

Worldwide, the number of migrants continues to increase [1]. This trend is reflected in Australia, with the proportion of overseas born residents increasing over the last decade [2] and reaching around a third (6.9 million people) of Australia’s population in 2016 [2]. Previous research indicates that migrants from low and middle income countries living in high income countries may have differing health and healthcare needs compared to the host population [3,4,5], including migrants in Australia [3,4]. An example of this is the higher rates of HIV among people born in sub-Saharan Africa (SSA) (13.1 per 100,000), Southeast Asia (SEA) (14.0 per 100,000) and Northeast Asia (NEA) (4.8 per 100,000) compared to Australian born (3.2 per 100,000) [6]. From 2013–2017, the proportion of late diagnoses for HIV (measured by a CD 4 cell count of fewer than 350 cells/µL at diagnosis) was highest amongst people born in sub-Saharan Africa (53%) and Southeast Asia (48%) [6]. Consequently, people from these regions have been recognised as priority populations in successive Australian HIV strategies [7]. 

The reasons for late diagnosis of HIV among SSA and SEA migrants living in high-income countries (HIC), such as Australia, are broadly understood. Our previous research indicated that population mobility (movement between countries) can be a risk factor for HIV, as risk behavior of the individual may change in the destination country [8,9,10,11,12,13]. Mobility may also increase vulnerability to HIV acquisition, as an individual’s access to health services and social support changes [8]. In Australia, migrants may have varying access to health services based on their visa status [8]. Additionally, migrants may have difficulty accessing healthcare services due to language barriers, and experiences of stigma and discrimination [14,15,16]. Migrants may also experience challenges in navigating the healthcare system, such as knowledge of how to make appointments, or what services are accessible to them [12,17,18,19,20]. An inadequate understanding of primary healthcare and preventative health, including cultural differences related to seeking healthcare, may also limit access [21,22].

Compounding barriers to accessing health services, there are also specific barriers to accessing sexual health services including: embarrassment, feeling ashamed, or fear of being judged by both community and health service providers [12,14,20,21,23,24]. Specifically for HIV, stigma has been identified as a barrier to testing [22], resulting in a low perception of individual risk (in that HIV is seen as ‘for others’) or fear of isolation from community [23]. A systematic review of barriers and facilitators to HIV testing among migrants in HIC also found low knowledge of HIV, poor patient-provider relationships, and concerns of confidentiality acted as barriers to testing [25]. There has been little research addressing Australian migrants’ perspectives on the barriers and facilitators to accessing HIV-related health services in Australia [13]. 

A cross-sectional survey of culturally and linguistically diverse (CaLD) communities in New South Wales (NSW) identified general practice as the most common location for HIV testing (59%), followed by sexual health clinics (15%) [26]. In Australia, HIV testing typically involves venipuncture specimen collection in medical settings. In 2012, rapid HIV testing (RHT) was made available in Australia, supported by the 2011 National HIV Testing Policy [27]. Previous research in Australia has identified RHT as a preferred testing mechanism for gay and bisexual men (GBM) [28,29,30]. The first self-test for HIV was recently approved for use in Australia, in November 2018 [31]. Australian research has showed support for self-testing among GBM [32], as well as increased frequency of testing, particularly among those who were non-recent (>2 years ago or never tested) testers [33]. 

Little is known about the acceptability of these new HIV testing technologies among people from SSA, SEA and NEA living in Australia [34]. RHT and self-testing may present an opportunity to increase HIV testing rates among migrant communities living in HIC like Australia. The aim of this study was to: (1) identify barriers and facilitators to HIV testing; and (2) explore knowledge and perceived acceptability of new HIV testing strategies, among people born in SSA and SEA/NEA living in Australia. 

## 2. Methods

This research sought to gain a better understanding of the barriers to HIV testing for people born in SSA and SEA/NEA through focus groups. It was part of a larger project that also included interviews with GPs on their perspectives of the barriers to testing patients from SSA and SEA/NEA for HIV [35]. The domains of the consolidated criteria for reporting qualitative research (COREQ) guided the development of this research and reporting on the findings [36]. 

### 2.1. Research Team and Steering Group

This research was conducted by two Australian born, public health researchers, both females: a recent university graduate project officer, supervised by a university-based academic with previous experience in community HIV work, qualitative research and working with mobile populations. Both members of the research team had previous experience in research with migrant communities and HIV. 

The research was overseen by a larger project steering group across several jurisdictions, consisting of researchers at three universities, a senior policy officer at a state department of health and a practice manager for a multicultural services organization. All steering group members have experience in qualitative research and working with migrant communities. The steering group provided overarching guidance on the development of the focus group discussion guide and on methodology and data analysis. The group met at the start of the research and members of the steering group were contacted separately (via phone, email and face-to-face) for assistance, depending on the expertise sought. 

### 2.2. Project Advisory Group

To ensure relevance of the research to the Western Australian (WA) sexual health and blood-borne virus sector, and the cultural appropriateness of the research, WA organizations (*n* = 6) were engaged to participate in a project advisory group. Group members worked in government and non-government organizations and were interested in migrant sexual health. The project advisory group helped develop the focus group discussion guide, determined the best locations to recruit participants through various methods and assisted in recruitment of participants (where possible). The group met at the start of the research and members of the project advisory group were subsequently contacted by the research team separately (via phone, email and face-to-face) for assistance, depending on the expertise sought. 

### 2.3. Focus Group Facilitators

Peer facilitators were sought, wherever possible, to help recruit for and conduct the focus group discussions. In this study, a peer facilitator refers to someone born in Africa or Asia who led the focus group discussions. The steering group and project advisory group assisted in recruiting six peer facilitators through various methods. The use of peer facilitators helped ensure the culturally appropriate delivery of focus groups, with a view to capture more useful and rich data [37]. Because of shared cultural characteristics, it has been posited that peer facilitators are likely to better develop rapport with participants [38]. Whilst recognising the influence of power dynamics between participants and peer facilitators related to gender, age, culture and community, it has been suggested that participants may be more to be willing to discuss shared experiences, given commonalities [38]. Indeed, it has been suggested that participants provide the best account to facilitators from the same culture [39]. For this research, focus groups were often co-facilitated by a peer facilitator and a non-peer facilitator from the research team in order to manage the potential power differential. 

To support peer facilitators and provide some consistency in delivery between facilitators, a guidebook was developed which included information about the research, focus group methodology and instructions for delivery. In particular, the guide focused on why focus groups were being used in the research, and gave instructions on how to facilitate focus groups, including managing participants, confidentiality and boundaries and ethical considerations including those around stress and support. Peer facilitators received additional support from a member of the research team as needed. A member of the research team was present at all focus groups to provide support to both peer facilitators and participants. An honorarium (AUD $100) was offered to peer facilitators to acknowledge the important role that they played in the success of the research. 

### 2.4. Participant Eligibility and Recruitment

Eligible focus group participants were adult (over 18 years old) males or females, SSA or SEA/NEA. Peer facilitators, as well as people who worked closely with the target group, developed targeted recruitment strategies. Recruitment of study participants occurred via advertisements on social media (Facebook), e-lists for multicultural services, at clinics for men who have sex with men (MSM), and through individuals’ networks.

### 2.5. Study Setting

Focus groups were conducted in the metropolitan areas of four Australian jurisdictions: Perth, Melbourne, Adelaide and Sydney in a range of venues, as suggested by the peer facilitators or steering group members. These venues included local multicultural community centers, local non-government centers and tertiary education institutes. The venues were chosen based on ease of access, if they were already known to participants, and where a quiet room inaccessible to the public was available. 

### 2.6. Methodology

This research used an interpretative phenomenological analysis (IPA) methodology in its conceptualization [40,41]. This approach attempts to understand the lived experience of participants and how they make sense of their experiences [42]. It includes elements of ‘giving voice’ and ‘making sense’ and has been used in other research with focus groups, in particular with CaLD communities [43,44]. 

Focus group discussions were chosen as the data collection method for their strengths in directly reflecting the voices of CaLD individuals and the ability to encourage participants to share, build on and discuss sensitive topics [45]. The synergistic effects from discussing and contributing ideas are key features of focus groups that cannot be achieved during individual interviews [46,47]. Focus groups also help to validate points raised as ‘shared’ experiences. Focus groups may also enable people who may be unable to read or write to participate; can encourage participants who may be reluctant to be interviewed on their own, and give insight into the groups’ range of lived experiences [46]. Focus groups have been used with CaLD communities in Australia, including in sexual health research [12]. 

### 2.7. Ethical Considerations

The Curtin University Human Research Ethics Committee approved this study (approval number HRE2017-0088), and was noted by the University of New South Wales. Ethical approval was also granted by the AIDS Council of New South Wales (ACON) Research Ethics Review Committee (RERC reference number: 2017/13). The involvement of the steering group and project advisory group in the project design ensured that the data collection methods and instruments used were culturally appropriate and safe. Peer facilitators were also invited to provide feedback on the study design. During focus group discussions, two trained facilitators explained the rationale for the study and the risks and benefits of participation. Participants were provided with an information sheet, which used simple English to support understanding; were asked to provide written consent; and were given the option to withdraw from the research at any point in time. 

To address difficulties commonly associated with discussing sensitive topics in focus groups, participants were informed that they did not need to contribute any personal information unless they were comfortable doing so [48]. Only first names (or pseudonyms) were used during the focus groups, and participants were asked not to share any experiences discussed by others after the focus group [37]. Focus groups included questions about knowledge of HIV, attitudes towards testing, and experiences of accessing health services [48]. A debriefing session was conducted after each focus group, in which the facilitators addressed any misinformation that came up during the focus group and answered any queries on the topic. Referrals to a range of sexual health services and contact details of the project officer were provided to participants. Data could only be accessed by the researchers and were stored securely in a university online repository [49]. Anonymity was maintained during data analysis; gender and country of birth (or region if country not identified) were only used to demonstrate the diversity of responses represented by exemplar quotes in reporting.

### 2.8. Focus Group Guide Development

A focus group guide was developed for the facilitators incorporating questions based on previous studies reported in the peer-reviewed literature, in line with the research objectives and with IPA methodology [41]. The focus group guide was semi-structured, to encourage facilitators to explore topics of interest as they arose. Questions were broad and open, and exploratory in nature [41]. The focus groups were designed to be informal and conversational in nature, providing an opportunity to understand participants’ terminology and world-view. A section of the focus group guide was pilot tested with a group of female postgraduate qualitative research students (*n* = 6) to gauge understanding and flow of the questions. Revisions were made as required to reflect feedback from the testing. 

### 2.9. Data Collection and Analysis

Eleven focus groups were conducted to capture diverse experiences among different groups. These involved: three discussions with MSM from SEA/NEA; three discussions with women from SEA/NEA; three discussions with people of mixed gender from SSA; one discussion with men from SSA and one discussion with women from SSA. The decision to conduct gender-specific, or mixed gender groups, for SSA groups was determined by the facilitator. The decision to include mixed gender groups was based on the facilitator’s knowledge of that community, and their perception of the group’s comfort with participating in a mixed gender discussion. 

The number of participants per group ranged from 4–12 individuals. All focus group discussions were audio-recorded with participants’ consent and each lasted between 45 and 90 min. Participant demographics were recorded via a self-administered paper-based questionnaire at the start of the focus group discussions. Participants were offered refreshments and an AUD$20 gift card to acknowledge their time. 

Audio recordings were transcribed verbatim in English by the lead researcher enabling immersion in the data [50]. Transcripts were checked against recordings for accuracy. Member checking occurred by sending transcripts to facilitators to check accuracy, and confirm the main themes occurring in the focus group. Thematic analysis was used as a process of identifying and reporting on patterns (or themes) from the data, using phases discussed by Braun and Clarke, 2006 [50,51]. Transcripts were read through once, then coded using NVivo software version 11 by the lead researcher [52]. Initial coding focused on the language and descriptions used by the participants, and on identifying the main concerns [40]. Coding was conducted line-by-line, and through open coding. Subsequently, codes were organized into core themes by grouping and combining similar codes. Literature was read concurrently throughout data analysis to better explain and further refine themes. Main themes were discussed and further refined with two members of the steering group. Verifications strategies used to ensure rigor included congruence between research question, literature, recruitment, data collection and analysis [53]; concurrent collection and analysis of data [53]; clear record keeping to establish an audit trail [53]; rich verbatim narratives from participants [54]; and involving other researchers to reduce researcher bias [54].

### 2.10. Demographics of Participants

Focus groups involved 77 participants, comprising 45 women and 31 men (Table 1). Half of the participants were aged between 25–34 years (*n* = 37), more than two-thirds had completed a university undergraduate degree or higher (*n* = 52) and a large proportion had lived in Australia for between one to five years (*n* = 21) (Table 1). Participants from SEA/EA countries were born in Indonesia (14), China (7), Malaysia (4), Philippines (3), Vietnam (3), India (2), Hong Kong (2), Myanmar (1), Pakistan (1), Sri Lanka (1), Taiwan (1), and Thailand (1) (Note: By Australia Bureau of Statistic Standard Australian Classification of Countries, India and Sri Lanka are classified as South Asia). SSA participants were born in South Sudan (19), Somalia (5), Botswana (2), Tanzania (2), Nigeria (2), Democratic Republic of the Congo (1), Djibouti (1), Ghana (1), Kenya (1), and Zambia (1).

## 3. Results

**Figure 1:**
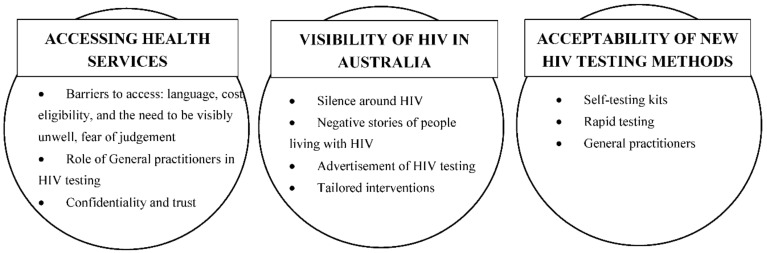
Core themes and subthemes.

Participants expressed a diverse range of experiences relating to sexual health and HIV testing. In this section, we describe the three core themes raised by SSA and SEA/NEA and participants. These themes were: (1) access to health services in Australia; (2) visibility of HIV in Australia and (3) acceptability of new testing methods (Figure 1). 

**Figure 2:**
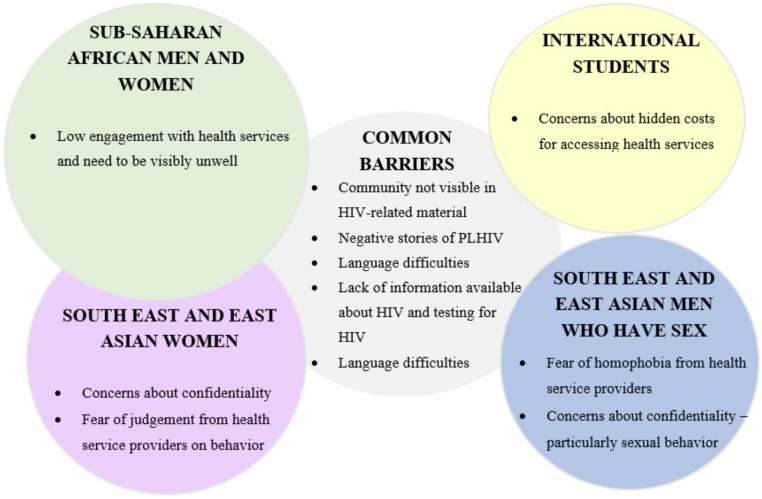
Barriers to HIV testing—commonalities and differences across groups.

There were distinct barriers to HIV testing for participants who were international students (both from SSA and SEA/NEA), or Asian MSM, as well as some variability between those from SSA and SEA/NEA. An overview of the common barriers, and barriers unique for each group, are shown below (Figure 2). 

Participants have been quoted verbatim, and gender, country of birth and the focus group participated in is reported on. Focus group questions asked about ‘community’ perceptions and experiences. For SSA men and women and SEA/NEA women, ‘community’ was mostly used to describe the people from their country of birth. However, for SEA/NEA MSM, the term ‘community’ was often used to describe the gay community that they had connected with in Australia, which depended on sexuality rather than country of birth.

### 3.1. Accessing Health Services

Most participants indicated that being able to access health services was important for them. Low levels of engagement with health services were described as limiting opportunities to test for HIV. Barriers raised included: cost and eligibility (for international students), the need to be visibly unwell (for SSA participants) and language difficulties (all groups). Most participants wanted GPs to offer HIV testing, but were concerned about confidentiality (for SEA/NEA MSM), and being discriminated against by GPs (for women from SEA/NEA). 

International students raised the issue of cost and ineligibility (not being perceived as being allowed to access) as the main barriers to accessing health services. While students are required to have Overseas Student Health Cover, there remained issues of cost. One African international student described the issue of cost as being “*fined to be sick”* when accessing health services. This deterred access to health services:

*“I came here like four years ago, I’m a Chinese student, so I’ve only been to GP like once because it’s extremely expensive.”*

M, China (Asian MSM group, international student)

Some participants described the Australian health care system as confusing, and expressed frustration with the process of having to book appointments (particularly when they were sick), or with perceived poor quality of the medical staff:

*“…they are probably too professional … your thing (illness) is so pressing, but you still have to go through that process of making appointments, while you have to feel the pain.”*

M, Botswana (African mixed gender group, international student)

For those from SSA who had arrived some years ago, many as refugees, experiences in their country of birth shaped willingness to access health services in Australia. Visiting the doctor was often believed to be a luxury. There was a perception that one needed to be visibly physically unwell, described as being on their “death bed” and that this sickness was recognizable to others, in order to access health services:

*“… (I’m) here for ten years and I don’t have malaria or anything, I don’t go to a doctor. So this is a common thing with us (African men), you only see the doctor when you are sick.”*

M, South Sudan (African male group)

Language difficulties were described as an issue for some in the community, and concerns were raised about the confidentiality of the interpreter. This was especially the case for small language groups in Australia, with few interpreters available. There were also issues relating to the gender of the interpreter given the often ‘taboo’ nature of sexual health. These language issues and challenges of using an interpreter presented difficulties for discussing sexual health with a GP:

*“We (have) been here, our generation over 6-10 years here and … we are still having language barriers that impacts mostly on those (sexual) health issues.”*

M, South Sudan (African male group)

#### 3.1.1. Role of General Practitioners in HIV Testing

Most participants regarded GPs as the gatekeeper of health-related knowledge, and this included HIV. Therefore, if HIV was not discussed by the GP, it was seen as a non-issue—particularly in instances where individuals had attended for a sexual health check-up, an overall health check-up or had their blood taken. A number of participants stressed that these experiences presented missed opportunities to be tested for HIV. Participants discussed instances where GPs had refused to provide an HIV test, having determined that individual not at risk. One participant recalled that the GP had refused her request for an HIV test, citing the fact that she was a woman:

*“…she (GP) doesn’t deem me to be in the high-risk category, but it would be nice to just have the (HIV) test done.”*

F, Malaysia (Asian female group)

Some participants expressed concern about experiencing discrimination by GPs. This was the case where participants felt that by being offered an HIV test, the GP was making an assumption about their behaviour. Instead, these participants indicated that HIV testing should be offered to everyone rather than only in relation to specific risk behaviours, to avoid having to disclose very personal information:

*“Because there is a certain judgement involved with the questions, your GP will (think) ‘Is she a slut? Is she a drug addict? How many men has she slept with?”*

F, Malaysia (Asian female group)

For some participants from Africa, there was a perception that HIV was seen by the broader community as an “African disease”. These participants stressed the importance of not targeting African communities, and for health services not to offer testing based on country of birth.

*“(HIV is) seen as an African disease… you go to the hospital and you say ‘hey doc, I think I’ve got HIV’, they might judge you.”*

F, Kenya (African women’s group, international student)

#### 3.1.2. Confidentiality and Trust

Some participants expressed concerns about doctor-patient confidentiality in Australia. While some participants indicated that they trusted that the information they disclosed and believed that the results of their test would be safe, others were concerned that confidentiality would be breached. Past experiences of a breach of confidentiality in countries of birth contributed to this concern. This was particularly the case for SEA/NEA MSM. These participants expressed concern that the government, community, or family, would know the result of an HIV test, the behavior that they engaged in or who they had had sex with. For these reasons, some participants recounted instances of not disclosing high-risk behaviors to their GP (i.e., not reporting sexual encounters outside of marriage, or sexual encounters with the same-sex):

*“…I had to hand over Medicare— would my parents be notified? Would they find out?... they say it’s anonymous, but they could track me, I just signed documents, my names on paper… it tells people I’m having sex (with men).”*

M, Indonesia (Asian MSM group)

### 3.2. Visibility of HIV in Australia

Participants commented that they had seen very little about HIV whilst living in Australia. For those from SSA and for MSM who had previously seen information on HIV (such as community workshops, pamphlets and online information), they reflected that this was often targeted towards Australian gay men. 

Overall, most participants wanted to see and hear more about HIV—positive stories of people living with HIV (PLHIV) in Australia, information on HIV that was relevant to them (for example, consequences of testing HIV positive for international students, practicing safe sex while returning to Africa to conceive) and relevant services for support if diagnosed positive. 

#### 3.2.1. Negative Stories of HIV

Some participants noted that they had only seen or heard negative information about HIV while in Australia. Some examples discussed included criminal cases of HIV transmission (predominantly relating to African men) in the media, cases of PLHIV not obtaining permanent residency, PLHIV ‘disappearing’ or being isolated from the community, or rumors about government quarantining PLHIV. These stories contributed to the belief that HIV was a taboo topic in Australia. This contributed to participants’ concerns that they would be judged for seeking information or requesting an HIV test. There were also concerns about PLHIV being removed from Australia:

“*I remember one of my friends asked “so what if you find HIV positive, what do you do?”…“you get locked up, isolated areas” …you find you’re HIV positive, you get isolated, you don’t stay in the community, that’s what they say, you don’t see your family and stuff.”*

M, South Sudan (African male group)

#### 3.2.2. Advertisement of HIV Testing

While some participants demonstrated a good understanding of HIV transmission, they lacked knowledge on where to test, which organizations to contact for information on HIV and testing, what testing involved, and what a positive result would mean. For some, the lack of information available on HIV contributed to feelings that HIV was not an issue in Australia—or that it was an issue that was not being dealt with:

*“Where do you test? …if I have problems, I see a physician here, I see a dentist….I haven’t seen any poster of HIV. No testing centres… nobody is dealing with the HIV.”*

M, Tanzania (African mixed gender group, international student)

Some participants (particularly international students and more recent arrivals to Australia), lacked knowledge on where to access health services for sexual health, particularly in locations where it was free:

*“You can’t share it (HIV knowledge) as freely as back home. Because at home (Kenya)… there is free testing. It’s unacceptable (that) in Australia it’s not free… when you google “free (sexually transmitted infections) places to test in Australia” it only brings up two tests—that’s gonorrhea and chlamydia.”*

F, Kenya (African women’s group)

#### 3.2.3. Tailored Interventions

Those participants who were more aware of Australia’s approach to HIV testing and treatment (mainly those from SSA who had lived in Australia for a number of years), commented that most HIV-related material such as pamphlets, information available online, etc. was targeted at gay men and there was a dearth of information tailored towards other communities. This resulted in difficulty accessing information that was seen as being appropriate (in terms of having information on risk factors and access to testing that was relevant to other communities) and as being culturally appropriate (mainly in that the images and language used did not reflect the community). Participants noted a need for information to be presented in a way that was relevant to their communities:

*“(We need) something about us, not about them (gay man).”*—

F, Africa (African mixed gender group)

For MSM who were not comfortable being connected with the gay community, the association of HIV with gay and bisexual men created barriers to accessing information. In addition, most HIV material was seen as being for ‘Australian gay men’, while gay men from other countries were perceived to be invisible in any promotional campaigns. Similar to the perceptions of the broader African and Asian communities, these participants wanted to see more material that represented men who were born overseas:

*“I think the international people they, they are quite lost in the message of HIV; it’s more Australian people.”*

M, China (Asian MSM group)

Likewise, other participants described the need for community to be actively engaged in the HIV response. For them, this meant the involvement of community and religious leaders, as well as representation of community in health services and organizations working in the HIV space. One African woman described people from outside her community coming in to deliver education without consultation and the challenges of cross-cultural communication:

*“They (community outsiders) just come in with a PowerPoint… it would be good to have more employed staff from similar cultures because they would understand how to communicate the message.”*

F, Africa (SSA mixed gender group)

### 3.3. Acceptability of New HIV Testing Methods

We asked participants about the acceptability of testing for HIV via GPs and sexual health clinics, rapid testing, and self-testing kits. Overall, there was a lack of knowledge about methods of HIV testing. While this was an anticipated finding for newer methods (i.e., rapid testing and self-testing), of concern was a lack of knowledge about testing for HIV via a GP, or a sexual health clinic, including among some MSM. For some participants, there was a perception that HIV testing was not available in Australia, again fueled by low visibility:

*“I didn’t even know you could go to the GP to test these things (HIV)… I’m thirty years old, and I didn’t even know this—it’s something so basic.”*

M, Indonesia (Asian MSM group)

Across all groups, there was a general consensus that having more options to test for HIV for different people and groups would only serve to increase testing. Specific benefits and concerns for each method are outlined below. 

#### 3.3.1. Self-Testing Kits

Participants raised concerns about the lack of immediate support for people who might receive a positive result (i.e., indication of HIV antibodies in sample) when using a HIV self-testing kit. In particular, it was suggested that people who believed HIV was a death-sentence, or feared exclusion from community, may not willingly seek a confirmatory test for HIV and be linked into care.

*“…I do it myself, after testing I am positive, what do I do? What is the next step?”*

F, Botswana (African mixed gender group)

Instead, some participants suggested that the purchase of self-testing kits (particularly oral versions) at GPs or pharmacy would provide more support. This way, a person could receive information from a health professional on what to do if they had a positive test, and had a place to go for support if the test was positive.

The benefits of self-testing kits were described mainly as confidentiality, including wanting to avoid the health system or Australian or country of birth government finding out they were testing for HIV. This was particularly important for MSM—several (all born in China) were already purchasing self-testing kits overseas. They described these as being easy to use, and a way of testing frequently:

*“I think most Chinese, especially gay people, prefer buying (a) HIV kit from the internet—it’s very convenient in China and it protects your privacy to some extent… and I will know the result in just two or three minutes.”*

M, China (Asian MSM group)

Self-testing was also of interest to participants, both males and females, who identified as heterosexual, for the privacy it offered. It was suggested that self-testing meant avoiding having to disclose information to a GP, and the challenges for some in openly describing their sexual behavior:

*“Myself, I prefer home testing because (I am) scared of telling GP (about sexual behavior) and would die of embarrassment.”*

M, South Sudan (African male group)

The option to do the test orally was suggested as a positive, particularly for women from SEA/EA who expressed a dislike of needles. Several individuals shared concerns about venipuncture collection, with some expressing concern about just how much blood was drawn for testing:

*“But I reckon it would take a lot of blood from the body every 3 months, sometimes they take like two syringes worth.”*

M, Asia (Asian MSM group)

#### 3.3.2. Rapid Testing

Rapid-testing was perceived to be the most time-efficient option and a useful method for improving testing frequency. This was suggested by participants to be particularly useful for those who were anxious and who did not wish to wait for results, or for those who were concerned about having a blood test:

*“(I) kind of, regret to take the (venipuncture HIV) test. ‘Oh my god why do I have to suffer like this?’ I want the rapid test, I want to make it fast especially if that’s my first test… I think about the different people I’ve been with over the seven days … ‘Am I positive, am I negative’ … (it) wasn’t a good (experience) for me.”*

M, Asia (Asian MSM group)

However, accuracy was described as the biggest concern for this method, particularly for those who were anxious about receiving a false positive result (someone without the virus receiving an incorrect positive result). 

#### 3.3.3. General Practitioners

Participants highlighted the important role of GPs in increasing HIV testing. Above all, participants suggested that they wanted HIV testing offered to them as part of a general health checkup:

*“(A) routine blood test… HIV (test) for general overall health, you know with blood pressure, cholesterol, the diabetes, the heart disease… it’s better us taking control over our own health… and then the HIV blood test becomes part of that as well.”*

F, Africa (African mixed gender group)

## 4. Discussion

People from SSA and SEA/NEA are overrepresented in HIV notifications in Australia [6,8]. Just under half of these notifications are diagnosed late [6]. This study contributes to a growing body of literature relating to migrants’ experiences regarding barriers to sexual health services, particularly HIV. Our research explored both barriers to HIV testing and the acceptability of new HIV testing methods for people born in SSA and SEA/NEA. A range of barriers, including sociocultural and structural factors, inhibited people from SSA and SEA/NEA accessing HIV testing. These barriers were mostly consistent with previous research [9,12,20,23,55,56] and systematic reviews [25,57,58,59,60]. This research adds a better understanding of the acceptability of novel modes of HIV testing in Australia, and reinforces the need for multi-strategic approaches to address HIV across individual, community, and policy levels.

### 4.1. Overview of Findings

One of the main barriers to HIV testing discussed in these focus groups was HIV-related stigma, experienced at an individual, community and policy level across all groups [61]. There was a perceived perception within communities that people living with HIV were “at fault” due to bad behaviour or being “bad” people—despite a reasonable knowledge of HIV transmission expressed among most participants and seen in previous Australian cross-sectional surveys [9,26,62]. For many, there was a fear of judgement if they were to test, particularly from health service providers. There were also concerns expressed about the confidentiality of both having the test, and the sexual behaviour disclosed, and whether this would be communicated back to partners, community members or family via health services. Social and cultural taboos around sexual behaviour and discussion of sex further limited sexual health help-seeking [21,63,64]. Anticipated experiences of stereotyping from health service providers, such as concern that HIV was seen “ also limited uptake of health services and HIV testing for SSA participants. International literature has found similar experiences among migrants, predominately from SSA. SSA migrants living in Belgium also described PLHIV as being at fault for their diagnosis and fear of stigma and social rejection limited uptake of testing [35]. SSA migrants in Ireland, the UK and New Zealand have described experiences of HIV-related stigma and racism from health service providers, where skin colour was perceived as being the proxy for HIV risk, rather than high-risk behaviour [21,22,36]. More broadly, research has reported on the negative impact that discrimination, and systemic discrimination, has on migrant help-seeking and health outcomes [12,65].

On a broader level, Australia’s current policies on immigration, particularly refugees, created a perception for some participants that “Australia has no HIV” due to compulsory HIV testing and rejected permanent residency applications of PLHIV [19]. “Silence” around HIV issues (low visibility of HIV information, e.g., media campaigns or pamphlets, or testing sites) contributed to this perception. Likewise, negative media stories of people living with HIV [66] were seen to support HIV-related stigma, particularly in criminal cases or cases that resulted in deportation [67] For some participants, a fear of deportation limited uptake of HIV testing, with previous Australian and international studies finding similar concerns among SSA and SEA/NEA migrants [12,19,22]. 

For many participants, resources on HIV (such as online media sources, pamphlets, posters, etc.) targeted Caucasian Australians, and participants noted that they did not see their communities (both ethnicity and sexuality) reflected in the images used. Given the unique role of culture, gender and social influences on individual perception of risk, and additional concerns of discrimination and racism, there is a need for community-specific approaches that address these factors [12,64]. It is important to recognize the heterogeneity of migrant groups and as far as is practicable to segment migrant groups in order to identify and respond to their unique needs [68,69]. This research identified a critical need for communities to see themselves represented in the HIV response while not reifying difference further or putting ‘blame’ on specific groups. 

In line with good health promotion practice, consultation and engagement with community is necessary to ensure that the community has buy-in to forthcoming projects and a voice on issues that most affect them [70]. Previous work has identified the role of community and religious leaders in addressing HIV stigma and increasing uptake of HIV testing [23,71], and peer workers in facilitating access to services [72]. To date, there is little published research on interventions to increase HIV testing among migrants in high-income countries [13,60], and further research is needed to identify what works with different groups in various settings. Interventions should target risks for migrants associated with frequent travel back to countries of birth, promote safe sexual behaviour whilst travelling, and encourage testing on return [26,64]. 

Consideration is required for testing offered in a setting and context that is comfortable to the needs of the individual. Participants stressed the need for a range of testing options, to provide additional opportunity for testing. For most participants (other than SEA/NEA MSM), there was a perception that GPs needed to do more to encourage uptake of HIV testing, including providing information about HIV and addressing concerns of confidentiality, treatment availability and implications of a positive result. Indeed, GPs play a vital role in ensuring early diagnosis of HIV and linkage into care in Australia, particularly among migrant communities who may not be accessing sexual health clinics [26]. Healthcare providers’ encouragement of HIV testing has been a successful method of increasing testing among migrant communities in Canada and the United States [58]. There are issues for engaging Australian mainstream GPs in encouraging testing uptake, including limited knowledge of HIV and a reliance on HIV specialist GPs to test for HIV [73]. While there are online training courses on HIV testing currently available for GPs in Australia [74], targeting GPs working predominantly with priority migrant communities to be involved in HIV testing, including messages of prevention [73], should be considered by peak bodies and state governments. 

Previous work has trialled self-testing kits [34], and rapid testing [75], with SSA and SEA/NEA migrants in Australia with encouraging levels of uptake. In our research, participants were generally accepting of these modes of testing, particularly in regards to the convenience offered and the reduced wait time for results. However, there were concerns about how to be linked into care in the case of a positive result. A recent systematic review on HIV testing for migrant communities in HIC also found rapid testing, testing outside traditional clinic settings and testing availability outside hours were positive interventions to increase testing [58]. Opportunities for outside of general practices should be explored by non-government organisations, and consideration of locations like community centres, sporting events, and cultural events may be testing effective [56,58]. Inclusion of other health screenings (e.g., blood pressure, glucose, etc.) may also work to normalise HIV testing among these communities [56]. 

### 4.2. Strengths and Limitations

There were some limitations to this study. Demographics of peer facilitators were not collected, and are unable to be reported. As the focus groups were conducted in English, without the option for an interpreter, findings may not reflect the perspectives of those with limited English proficiency. Further, the majority of participants had a University degree, and these findings may not reflect those without higher education. Given the sensitive nature of this topic, there was the possibility of social desirability bias, and differing experiences or opinions may not have been shared in the group setting [76]. It is also acknowledged that there are a number of characteristics that may influence whether an individual is considered a peer (such as gender age or class), that may influence trust and power dynamics between peer facilitators and participants [77,78]. 

People born in Indonesia and South Sudan were more frequently represented, and their experiences may be different to those from other countries. Whilst a strength, the inclusion of four jurisdictions may have masked differences in experiences between jurisdictions, such as service availability and connection to community. 

Many participants in the MSM focus groups were connected to the gay community and were frequently testing for HIV. Their levels of knowledge were generally higher than that of others and they had greater familiarity with services and testing procedures. This differed to the experience of a number of other participants who had only recently tested for HIV for the first time, and for those who had not yet tested—though these numbers were small overall. As such, the barriers for those who were more or less connected to community and services may have differed. 

This research had a number of strengths. The inclusion of participants from four Australian jurisdictions increased the relevance of this research nationally. Peer facilitators, working with non-peer co-facilitators, were able to draw out unique experiences between groups and as a secondary positive outcome, increase their own cultural competency. Future work will continue to build on the strength of involving community members as part of the research design and implementation. 

We included participants with diverse experiences—including people on temporary visas, MSM, and people across different age groups. Migrants are often conceptualized as a homogenous group, resulting in generalizations regarding health care needs [69]. Our research has attempted to identify possible similarities and differences between sub-groups of migrants to provide a better understanding of the barriers to HIV testing for diverse migrant groups. We acknowledge that there are additional sub groups and further variation within regions and countries that is not reported on here and which justifies further investigation [68]. 

## 5. Conclusions

These findings demonstrate the complexity and multifaceted nature of barriers to HIV testing for migrants. As such, interventions to address low levels of HIV testing among SSA and SEA/NEA migrants in Australia need to be multi-strategic and aimed at the individual, community and policy level. These findings have implications for policy-makers and for those working in practice. Australia has a goal of zero new HIV notifications by 2022—to achieve this it is critical that priority populations are engaged in regular HIV testing. New methods of HIV testing, including self-testing and rapid HIV testing, present an opportunity to engage with migrants outside of traditional health care settings. 

## Figures and Tables

**Table 1:** Socio-demographics of focus group participants by region of birth.

Characteristics	Number of Participants		
	Sub-Saharan Africa (*n* = 35)	Southeast Asia and Northeast Asia (*n* = 42)	All Participants (*n* = 77)
Sex *			
Male	12	19	31 (40%)
Female	22	23	45 (60%)
Age ** (years)			
18–24	6	5	11 (15%)
25–29	2	22	24 (32%)
30–34	6	7	13 (18%)
35–39	3	4	7 (9%)
40–44	7	2	9 (12%)
45+	11	2	13 (18%)
**Years lived in Australia**			
<1 year	6	11	17 (22%)
1–5 years	6	15	21 (27%)
6–10 years	3	8	11 (14%)
>10 years	20	8	28 (36%)
Status in Australia ***			
Citizen	23	9	32 (43%)
Permanent resident	4	6	10 (13%)
Student visa	6	22	29 (39%)
Other temporary visa	0	5	5 (7%)
Education Level ****			
Primary school	4	0	4 (5%)
High school	6	1	7 (9%)
Year 12 or equivalent	4	2	6 (8%)
TAFE certificate/diploma	4	2	6 (8%)
University postgraduate degree or higher	15	37	52 (69%)
Sexual identity *****			
Heterosexual	18	21	39 (54%)
Homosexual	1	17	18 (25%)
Bisexual	0	2	2
Pansexual	0	1	1
Other (not disclosed)	4	1	5 (7%)

Missing participant data—numbers do not add up to total: One sex missing *; Three ages missing **; Two statuses missing ***; Two levels of education missing ****; Five sexual orientation missing *****.

## References

[1] United Nations (2017). International Migration Report 2017 (ST/ESA/SER.A/403).

[2] Australian Bureau of Statistics 3412.0 - Migration, Australia, 2015-16. http://www.abs.gov.au/ausstats/abs@.nsf/mf/3412.0/.

[3] Stewart D.E., Do B.N. (2003). Health needs of migrant Vietnamese women in south-west Brisbane: An exploratory study. Aust. J. Soc. Issues.

[4] Mengesha Z.B., Perz J., Dune T., Ussher J. (2017). Refugee and migrant women’s engagement with sexual and reproductive health care in Australia: A socio-ecological analysis of health care professional perspectives. PLoS ONE.

[5] Ross J., Cunningham C.O., Hanna D.B. (2018). HIV outcomes among migrants from low-income and middle-income countries living in high-income countries: A review of recent evidence. Curr. Opin. Infect. Dis..

[6] The Kirby Institute (2018). HIV, viral hepatitis and sexually transmissible infections in Australia: Annual Surveillance Report 2018.

[7] Department of Health Australian Government (2014). Seventh National HIV Strategy 2014 - 2017.

[8] Crawford G., Lobo R., Brown G., Langdon P. (2014). HIV and Mobility in Australia: Road Map for Action.

[9] Gray C., Crawford G., Reid A., Lobo R. (2018). HIV knowledge and use of health services among people from South East Asia and sub-Saharan Africa living in Western Australia. Health Promot. J. Aust..

[10] Crawford G., Lobo R., Brown G., Macri C., Smith H., Maycock B. (2016). HIV, Other Blood-Borne Viruses and Sexually Transmitted Infections amongst Expatriates and Travellers to Low-and Middle-Income Countries: A Systematic Review. Int. J. Environ. Res. Public Health.

[11] Crawford G., Lobo R., Brown G., Maycock B. (2016). The influence of population mobility on changing patterns of HIV acquisition: Lessons for and from Australia. Health Promot. J. Aust..

[12] Agu J., Lobo R., Crawford G., Chigwada B. (2016). Migrant Sexual Health Help-Seeking and Experiences of Stigmatization and Discrimination in Perth, Western Australia: Exploring Barriers and Enablers. Int. J. Environ. Res. Public Health.

[13] Rade D., Crawford G., Lobo R., Gray C., Brown G. (2018). Sexual Health Help-Seeking Behavior among Migrants from Sub-Saharan Africa and South East Asia living in High Income Countries: A Systematic Review. Int. J. Environ. Res. Public Health.

[14] Drummond P.D., Mizan A., Brocx K., Wright B. (2011). Barriers to accessing health care services for West African refugee women living in Western Australia. Healthc. Women Int..

[15] Drummond P.D., Mizan A., Wright B. (2008). HIV/AIDS knowledge and attitudes among West African immigrant women in Western Australia. Sex. Health.

[16] Korner H. (2007). Late HIV diagnosis of people from culturally and linguistically diverse backgrounds in Sydney: The role of culture and community. AIDS Care.

[17] Åkerman E., Essén B., Westerling R., Larsson E. (2017). Healthcare-seeking behaviour in relation to sexual and reproductive health among Thai-born women in Sweden: A qualitative study. Cult. Health Sex..

[18] Thomas F., Aggleton P., Anderson J. (2010). “If I cannot access services, then there is no reason for me to test”: The impacts of health service charges on HIV testing and treatment amongst migrants in England. AIDS Care.

[19] Korner H. (2007). ‘If I had my residency I wouldn’t worry’: Negotiating migration and HIV in Sydney, Australia. Ethn. Health.

[20] Lindkvist P., Johansson E., Hylander I. (2015). Fogging the issue of HIV - Barriers for HIV testing in a migrated population from Ethiopia and Eritrea. BMC Public Health.

[21] McMichael C., Gifford S. (2009). “It is good to know now...before it‘s too late“: Promoting sexual health literacy amongst resettled young people with refugee backgrounds. Sex. Cult. Interdiscip. Q..

[22] Manirankunda L., Loos J., Alou T.A., Colebunders R., Nöstlinger C. (2009). “It’s better not to know“: Perceived barriers to HIV voluntary counseling and testing among sub-Saharan African migrants in Belgium. Aids Educ. Prev..

[23] Adedimeji A.A., Asibon A., O’Connor G., Carson R., Cowan E., McKinley P., Leider J., Mallon P., Calderon Y. (2015). Increasing HIV testing among African immigrants in ireland: Challenges and opportunities. J. Immigr. Minor. Health.

[24] Ussher J.M., Rhyder-Obid M., Perz J., Rae M., Wong T.W., Newman P. (2012). Purity, privacy and procreation: Constructions and experiences of sexual and reproductive health in Assyrian and Karen women living in Australia. Sex. Cult. Interdiscip. Q..

[25] Blondell S.J., Kitter B., Griffin M.P., Durham J. (2015). Barriers and Facilitators to HIV Testing in Migrants in High-Income Countries: A Systematic Review. AIDS Behav..

[26] McGregor S., Mlambo E., Gunaratnam P., Wilson D., Guy R. (2017). HIV knowledge, Risk Behaviour and Testing: A community Survey in People from Culturally and Linguistically Diverse (CALD) Backgrounds in NSW, Australia.

[27] National HIV Testing Policy Expert Reference Committee (2011). National HIV Testing Policy, 2011.

[28] Chen M.Y., Bilardi J.E., Lee D., Cummings R., Bush M., Fairley C.K. (2010). Australian men who have sex with men prefer rapid oral HIV testing over conventional blood testing for HIV. Int. J. Std. AIDS.

[29] Conway D.P., Guy R., Davies S.C., Couldwell D.L., McNulty A., Smith D.E., Keen P., Cunningham P., Holt M. (2015). Rapid HIV testing is highly acceptable and preferred among high-risk gay and bisexual men after implementation in Sydney sexual health clinics. PLoS ONE.

[30] Chan D., Stewart M., Smith M., Price T., Lusk J., Ooi C., Read P., Finlayson R. (2015). The rise of targeted HIV oral rapid testing in Australia. Med. J. Aust..

[31] Australian Federation of AIDS Organisations Getting tested for HIV. https://www.afao.org.au/about-hiv/getting-tested-for-hiv/.

[32] Bilardi J.E., Walker S., Read T., Prestage G., Chen M.Y., Guy R., Bradshaw C., Fairley C.K. (2013). Gay and Bisexual Men’s Views on Rapid Self-Testing for HIV. AIDS Behav..

[33] Jamil M.S., Prestage G., Fairley C.K., Grulich A.E., Smith K.S., Chen M., Holt M., McNulty A.M., Bavinton B.R., Conway D.P. (2017). Effect of availability of HIV self-testing on HIV testing frequency in gay and bisexual men at high risk of infection (FORTH): A waiting-list randomised controlled trial. Lancet HIV.

[34] Driver G., Debattista J., Gu Z., Lemoire J., Hooper J. (2017). HIV testing within the African community using home-based self collection of oral samples. Aust. N. Z. J. Public Health.

[35] Collaboration for Evidence Research and Impact in Public Health (2018). “I Want to Test but I’m Afraid”: Barriers to HIV Testing among People Born in South East Asia and Sub-Saharan Africa: Final Report.

[36] Tong A., Sainsbury P., Craig J. (2007). Consolidated criteria for reporting qualitative research (COREQ): A 32-item checklist for interviews and focus groups. Int. J. Qual. Healthc..

[37] Halcomb E.J., Gholizadeh L., DiGiacomo M., Phillips J., Davidson P.M. (2007). Literature review: Considerations in undertaking focus group research with culturally and linguistically diverse groups. J. Clin. Nurs..

[38] Liamputtong P., Liamputtong P. (2008). Doing Research in a Cross-Cultural Context: Methodological and Ethical Challenges. Doing Cross-Cultural Research: Ethical and Methodological Perspectives.

[39] Adamson J., Donovan J.L. (2002). Research in Black and White. Qual. Health Res..

[40] Palmer M., Larkin M., de Visser R., Fadden G. (2010). Developing an Interpretative Phenomenological Approach to Focus Group Data. Qual. Res. Psychol..

[41] Smith J.A., Osborn M. (2004). Interpretative phenomenological analysis. Doing Soc. Psychol. Res..

[42] Husserl E. (1990). Ideas Pertaining to a Pure Phenomenology and to a Phenomenological Philosophy: Second Book Studies in the Phenomenology of Constitution.

[43] Kuljit H., Michael L., Ivan B., John R. (2012). The cultural context of care-giving: Qualitative accounts from South Asian parents who care for a child with intellectual disabilities in the UK. Adv. Ment. Health Intellect. Disabil..

[44] Walls J.K., Hall S.S. (2017). A focus group study of African American students’ experiences with classroom discussions about race at a predominantly White university. Teach. High. Educ..

[45] Cox C.M., Babalola S., Kennedy C.E., Mbwambo J., Likindikoki S., Kerrigan D. (2014). Determinants of concurrent sexual partnerships within stable relationships: A qualitative study in Tanzania. BMJ Open.

[46] Culley L., Hudson N., Rapport F. (2007). Using focus groups with minority ethnic communities: Researching infertility in British South Asian communities. Qual. Health Res..

[47] Dias S., Gama A., Rocha C. (2010). Immigrant women’s perceptions and experiences of health care services: Insights from a focus group study. J. Public Health.

[48] Colucci E., Liamputtong P. (2008). On the Use of Focus Groups in Cross-Cultural Research. Doing Cross-Cultural Research: Ethical and Methodological Perspectives.

[49] National Health and Medical Research Council National Statement on Ethical Conduct in Human Research (2007). https://www.nhmrc.gov.au/guidelines-publications/e72.

[50] Braun V., Clarke V. (2006). Using thematic analysis in psychology. Qual. Res. Psychol..

[51] Brocki J.M., Wearden A.J. (2006). A critical evaluation of the use of interpretative phenomenological analysis (IPA) in health psychology. Psychol. Health.

[52] QSR International Pty Ltd (2016). Vivo Qualitative Data Analysis Software. Version 11.

[53] Morse J.M., Barrett M., Mayan M., Olson K., Spiers J. (2002). Verification Strategies for Establishing Reliability and Validity in Qualitative Research. Int. J. Qual. Methods.

[54] Noble H., Smith J. (2015). Issues of validity and reliability in qualitative research. Evid. Based Nurs..

[55] Shangase P., Egbe C.O. (2014). Barriers to Accessing HIV Services for Black African Communities in Cambridgeshire, the United Kingdom. J. Community Health.

[56] Bova C., Nnaji C., Woyah A., Duah A. (2016). HIV Stigma, Testing Attitudes and Health Care Access Among African-Born Men Living in the United States. J. Immigr. Minority Health.

[57] Deblonde J., De Koker P., Hamers F.F., Fontaine J., Luchters S., Marleen T. (2010). Barriers to HIV testing in Europe: A systematic review. Eur. J. Public Health.

[58] Alvarez-del Arco D., Monge S., Azcoaga A., Rio I., Hernando V., Gonzalez C., Alejos B., Caro A.M., Perez-Cachafeiro S., Ramirez-Rubio O. (2013). HIV testing and counselling for migrant populations living in high-income countries: A systematic review. Eur. Public Health.

[59] Mengesha Z.B., Dune T., Perz J. (2016). Culturally and linguistically diverse women’s views and experiences of accessing sexual and reproductive health care in Australia: A systematic review. Sex. Health.

[60] Aung E., Blondell S.J., Durham J. (2017). Interventions for Increasing HIV Testing Uptake in Migrants: A Systematic Review of Evidence. AIDS Behav..

[61] Stangl A.L., Lloyd J.K., Brady L.M., Holland C.E., Baral S. (2013). A systematic review of interventions to reduce HIV-related stigma and discrimination from 2002 to 2013: How far have we come?. J. Int. Aids Soc..

[62] Dean J., Mitchell M., Stewart D., Debattista J. (2017). Sexual health knowledge and behaviour of young Sudanese Queenslanders: a cross-sectional study. Sex. Health.

[63] Rogers C., Earnest J. (2014). A cross-generational study of contraception and reproductive health among Sudanese and Eritrean women in Brisbane, Australia. Healthc. Women Int..

[64] Mullens A.B., Kelly J., Debattista J., Phillips T.M., Gu Z., Siggins F. (2018). Exploring HIV risks, testing and prevention among sub-Saharan African community members in Australia. Int. J. Equity Health.

[65] Viruell-Fuentes E.A., Miranda P.Y., Abdulrahim S. (2012). More than culture: Structural racism, intersectionality theory, and immigrant health. Soc. Sci. Med..

[66] Persson A., Newman C. (2008). Making monsters: Heterosexuality, crime and race in recent Western media coverage of HIV. Sociol. Health Illn..

[67] Byrne E. (2016). Godfrey Zaburoni, who infected partner with HIV, has conviction quashed by High Court. ABC News.

[68] Ogilvie L.D., Burgess-Pinto E., Caufield C. (2008). Challenges and approaches to newcomer health research. J. Transcult. Nurs..

[69] Laverack G. (2018). ‘Leaving No One Behind’: The Challenge of Reaching Migrant Populations. Challenges.

[70] Hulse G.K. (1997). Australia’s public health response to HIV and HCV: A role for ‘affected’ communities. Drug Alcohol Rev..

[71] Australian Federation of AIDS Organisations (2014). HIV and Stigma in Australia: A Guide for Religious Leaders.

[72] Navarro M., Navaza B., Guionnet A., López-Vélez R. (2018). Overcoming Barriers to HIV Prevention and Healthcare Among Sub-Saharan African Migrants in Spain. JMIR Public Health Surveill..

[73] Newman C.E., Kidd M.R., Kippax S.C., Reynolds R.H., Canavan P.G., de Wit J.B.F. (2013). Engaging nonHIV specialist general practitioners with new priorities in HIV prevention and treatment: Qualitative insights from those working in the field. Sex. Health.

[74] Australasian Society for HIV, Viral Hepatitis and Sexual Health HIV Training. https://www.ashm.org.au/HIV/training/.

[75] Mutch A.J., Lui C.W., Dean J., Mao L., Lemoire J., Debattista J., Howard C., Whittaker A., Fitzgerald L. (2017). Increasing HIV testing among hard-to-reach groups: Examination of RAPID, a community-based testing service in Queensland, Australia. BMC Health Serv. Res..

[76] Hollander J.A. (2004). The Social Contexts of Focus Groups. J. Contemp. Ethnogr..

[77] Gabriel P., Kaczorowski J., Berry N. (2017). Recruitment of Refugees for Health Research: A qualitative study to add refugees’ perspectives. Int. J. Environ. Res. Public Health.

[78] Eklöf N., Hupli M., Leino-Kilpi H. (2017). Planning focus group interviews with asylum seekers: Factors related to the researcher, interpreter and asylum seekers. Nurs. Inq..

